# Exploiting cellular senescence in hematologic malignancies

**DOI:** 10.1016/j.tranon.2026.102729

**Published:** 2026-03-16

**Authors:** Peijie Jiang, Guancui Yang, Jiarun Li, Xiaolong Tian, Xueqing Yang, Shijie Yang, Jin Wei, Xi Zhang, Jinyi Liu

**Affiliations:** a Chongqing Key Laboratory of Hematology and Microenvironment, Medical Center of Hematology, Second Affiliated Hospital, Army Medical University, Chongqing 40037, China; b Department of Hematology, Affiliated Hospital of North Sichuan Medical college, Nanchong, Sichuan Province 637002, China

**Keywords:** Senescence, Hematologic malignancies, Therapy

## Abstract

Cellular Senescence is characterized by stable cell cycle arrest and major changes in cell morphology and physiology. Accumulating evidence indicates that senescence-associated phenotypic alterations in blood cells drive the initiation and progression of hematologic malignancies. Therefore, we reviewed the phenotypes associated with cellular senescence and the regulatory mechanisms of cellular senescence in hematological malignancies. Notably, chemical induction of cellular senescence and selectively elimination of senescent cells can effectively inhibit tumor cell proliferation and enhance the efficacy of chemotherapy. This review also discusses the role of cellular senescence in the treatment of hematological malignancies, providing an in-depth understanding of the role of cellular senescence as a double-edged sword in cancer biology.

## Highlights

• Characteristics of Senescent Cells: Morphological alterations, cell cycle arrest, DNA damage, SASP, SA-β-gal and lipofuscin, metabolic changes.
• Therapeutic strategies targeting cellular senescence: inducing cellular senescence and eliminating senescent cells.
• Combining current antitumor treatments and senolytics can eliminate senescent cells more effectively and improve treatment outcomes in hematologic malignancies.

## Graphical abstract

**Figure:**
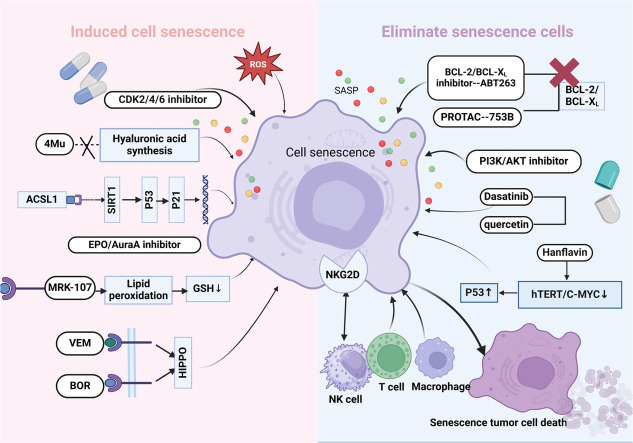

## Introduction

Cellular senescence refers to a stable state of cell cycle arrest, usually induced by DNA damage or oncogene activation [1]. In addition to cell cycle exit, cellular senescence is characterized by phenotypic changes, such as a senescence-associated secretory phenotype (SASP) and metabolic reprogramming [2]. Cellular senescence is commonly regarded as a tumor-suppressive mechanism that can inhibit the proliferation of tumor cells [3]. However, increasing evidence suggests that therapy-induced senescence can promote epithelial-mesenchymal transition (EMT) and tumorigenesis in neighboring cells [4], as well as re-entry into the cell cycle and activation of tumor stem cells [5], thereby increasing the survival of tumor cells [6]. Therefore, it is essential to eliminate therapy-induced cellular senescence. Here, we review the features of cellular senescence and the regulatory mechanisms of senescence in hematological malignancies and discuss therapeutic strategies targeting cellular senescence under these conditions.

## Characteristics of senescent cells

### Morphological and structural changes

Morphological alterations in cells are frequently observed in association with senescence [7]. During cellular senescence, cells exhibit increased volume, a flattened and irregular morphology, nuclear membrane invagination, nucleolar hypertrophy, chromatin remodeling, telomere attrition, and reduced nuclear structural stability [8]. Nuclear integrity is compromised owing to the loss of Lamin B1. Furthermore, the Rho GTPases Rac1 and CDC42 were found to be highly activated in senescent cells [9]. Moreover, vacuolization is associated with endoplasmic reticulum stress induced by the unfolded protein response [10].

### Cell cycle arrest

Cell cycle arrest is a definitive characteristic of senescent cells. Compared with quiescent cells, senescent cells do not respond to mitogenic or growth factor stimuli and cannot re-enter the cell cycle even when favorable growth conditions are present. The cell cycle arrest observed in senescent cells is mediated by the activation of cyclin-dependent kinase (CDK) inhibitors 1 and 2A (p21^CIP1^ and p16^INK4a^), which regulate the retinoblastoma protein (Rb) tumor pathway [11]. These inhibitors block the formation of cyclin-CDK complexes, which are involved in the G1-S phase transition at the cell cycle checkpoint [12]. CDKs phosphorylate various members of the Rb family, resulting in the release and subsequent activation of the G1-S phase transcription factor E2F. Activation of p53 induces the activation of p21^CIP1^, which blocks CDK2-cyclin E activity, thereby maintaining Rb in its low-phosphorylated G1 form and inhibiting the transcription of E2F target genes. However, E2F target genes are typically involved in DNA replication, promoting entry into the S phase, and they are further stabilized by Rb-mediated repressive chromatin marks. Consequently, the expression of E2F target genes is effectively repressed, resulting in cells remaining in prolonged G1 phase arrest. Furthermore, following p53 activation, Rb binds to retinoblastoma-like proteins 1 and 2 (p107 and p130), thereby inhibiting the transcription of associated genes and promoting cell cycle arrest and senescence [13]. p16^INK4a^ inhibits CDK4 and CDK6, resulting in prolonged cell cycle arrest. p16^INK4a^ is currently considered one of the most common and stable markers of cellular senescence. Consequently, when cells are exposed to stress, p21^CIP1^ and p16^INK4a^ are persistently activated to inhibit CDKs, maintain Rb in a low-phosphorylated state, and neutralize E2F transcription factors, thereby preventing the cell from entering the S phase.

### DNA damage

Cellular senescence and growth arrest are usually followed by a persistent DNA damage responses (DDR), which may result from intrinsic factors (such as oxidative damage, telomere shortening, and excessive proliferation) or extrinsic factors (such as ultraviolet light, γ-radiation, and chemotherapeutic agent exposure) [14]. DNA double-strand breaks (DSBs) are the most common triggers of the DDR. DNA DSBs promote the recruitment of ataxia-telangiectasia mutated (ATM) kinase, which binds to the DNA damage site [15]. This recruitment facilitates the phosphorylation of histone H2AX at Ser139 and increases the deposition of p53-binding protein 1 (53BP1), thereby promoting the assembly of specific DNA repair complexes. In addition, histone methylation promotes the assembly of specific DNA repair complexes. A complex containing transcription intermediary factor 1β, Rb-binding heterochromatin protein 1 (HP1) isoform, and the H3K9 methyltransferase SUV39H1 is loaded onto chromatin at DNA DSBs, leading to Rb-dependent methylation of histone H3K9 and the production of H3K9me3 as a senescence marker. In the early stages of DNA damage, ATM kinase-mediated DNA damage requires H3K9 methylation, with the final product H3K9me3 serving as a marker for senescence-associated heterochromatic foci near the promoters of E2F target genes [16]. Therefore, cellular senescence is associated with persistent DDR, leading to irreparable DNA damage. Additionally, the DDR associated with replicative senescence is telomere-dependent and is related to telomere uncapping and the overall loss of telomere length [15]. During oncogene-induced senescence (OIS), the occurrence of the DDR is unrelated to telomere length but is associated with telomere dysfunction. Although the DDR plays a role in initiating senescence, the role of DDR markers in identifying senescent cells in vivo remains unclear.

### SASP

Senescent cells produce a complex mixture of soluble and insoluble factors, collectively referred to as the SASP. The SASP refers to the total number of cytokines, chemokines, extracellular matrix proteases, growth factors, and other signaling molecules secreted by senescent cells. One regulator of the SASP is the DDR, a signaling cascade that senses and ultimately repairs DNA damage [17].

The main components of the SASP include the p38MAPK, mTOR, NF-κB, and C/EBPβ signaling pathways. p38MAPK is a member of the mitogen-activated protein kinase (MAPK) family. p38MAPK is activated by the phosphorylation of tyrosine and threonine in response to genotoxic stress (e.g., oncogenic RAS) during the senescence response [18], which leads to DNA damage [19]. Moreover, p38MAPK has been reported to regulate NF-κB activity in senescent cells in an induced senescence model. In addition, p53 suppresses the SASP by limiting p38MAPK activation, and p38MAPK activation occurs independently of the DDR [20]. mTOR-mediated phosphorylation of the 4EBP translation inhibitor protein regulates interleukin-1α (IL-1α) [21], and MAP kinase-activated protein kinase 2 (MAPKAPK2) [22] inhibits ZFP36L1, which is an mRNA-binding protein that specifically targets the mRNA degradation of proinflammatory SASP components. Therefore, mTOR can control senescence secretory activity by indirectly regulating the stability of SASP mRNAs [22]. NF-κB and C/EBPβ are partially activated in the chromatin of senescent cells [[23], [24], [25]] and directly regulate the transcription of key regulators of the inflammatory SASP, such as interleukin-8 (IL-8) and interleukin-6 (IL-6), to regulate the components of the SASP. Moreover, IL-6 and IL-8 play roles in an autocrine feedback loop to enhance the activity of C/EBPβ and NF-κB, thereby amplifying SASP signaling [23,25]. SASP factors are the primary paracrine signaling messengers between senescent cells and their surrounding cells, including stromal by stander cells, immune cells, precancerous cells, and cancer cells. For example, senescent cells signal to neighboring cells and influence them through mechanisms such as adjacent NOTCH/JAG1 signaling [26], ROS production [27], cytoplasmic bridge formation [28], or exosome release [29]. The SASP has both beneficial and detrimental effects on organisms. For example, the SASP prevents senescence by downregulating the expression of IL-6R, insulin-like growth factor binding protein 7 (IGFBP7), or IL-8 and related chemokine receptors (CXCRs) through autocrine regulation [23,25,30]. This autocrine mechanism contributes to the suppression of senescent tumors. The SASP of senescent hepatic stellate cells (HSCs) promotes the proliferation and malignancy of surrounding liver cells in obese mice treated with chemical carcinogens [31]. However, the SASP also mediates the harmful effects of accumulated senescent cells in vivo during chemotherapy [32]. Recent studies have suggested that the detrimental effects of the SASP may outweigh its beneficial effects. Moreover, the SASP is not a unique component of senescence. The specific SASP components that drive the functional aspects of the SASP in specific in vivo environments can only be elucidated through staining or single-cell analysis. Therefore, the application of the SASP as a biomarker is limited. Emerging evidence indicates that indoxyl sulfate induces cellular senescence via activation of the ROS-NF-κB-p53 signaling axis [33]. This process is mechanistically linked to the DDR, which activates NF-κB and promotes the secretion of SASP factors in senescent cells [34]. However, contrasting findings from those of an independent study have demonstrated that the NF-κB2/RelB heterodimer exerts senescence-modulatory effects on primary human fibroblasts through a distinct mechanism involving: (1) regulation of RB activity and (2) EZH2-mediated epigenetic repression of p21 and p53 [35].

### Lysosomal dependent changes

Senescence‑associated β‑galactosidase (SA-β-gal) is the most widely used senescence marker. It is active in normal cellular environments under physiological conditions (pH 4.0–4.5). In senescent cells, the activity of SA-β-gal increases because of the elevated lysosomal content [36,37]. Therefore, performing a histochemical assay for SA-β-gal activity in a cellular environment at pH 6.0 (the suboptimal pH for normal cells) allows for the specific identification of senescent cells [38]. In senescent cells, SA-β-gal activity originates from lysosomal β-galactosidase. Cells lacking the gene encoding lysosomal β-gal (GLB1) exhibit significantly reduced SA-β-gal activity, but this reduced activity does not interfere with cellular senescence [36]. Therefore, the increase in SA-β-gal activity in senescent cells is a result of aging rather than a cause of aging. α-Fucosidase, another lysosomal enzyme is also a biomarker of aging [39]. The increased lysosomal content in senescent cells may increase autophagy [40]. This increased autophagy is a characteristic of active macrophages, Kupffer cells, and osteoclasts [41,42]. Therefore, the use of other markers in conjunction with the monitoring of SA-β-gal activity is necessary to identify senescence. Lipofuscin, an aggregate of oxidized proteins, lipids, and metals, accumulates in aging tissues and colocalizes with SA-β-gal activity in senescent cells [43]. Increased SA-β-gal activity and lipofuscin activity are essential markers of senescent cells, reflecting lysosomal and autophagy-related abnormalities and mTOR signaling dysregulation. mTORC1/S6K1 activates the RAS signaling pathway and upregulates the expression of cell cycle inhibitors, such as p53, p21, and p16, ultimately leading to cellular senescence[[44], [45], [46], [47]]. In endothelial senescence, the mTORC1-S6K1 signaling pathway plays a pathogenic role in the uncoupling of endothelial nitric oxide synthase [48] and the expression of coagulation factor tissue factor [49,50]. Additionally, mTOR regulates the translation of MAPKAPK2, which inhibits the SASP [22]. However, the impact of mTORC2 on endothelial cell senescence remains controversial. Studies have shown that the mTORC2/Akt/GSK-3β/C/EBPα/Nrf2 signaling pathway is involved in replicative and induced endothelial cell senescence [51]. In an in vitro model of induced senescence with PTEN deficiency, the activation of mTORC1 or mTORC2 has been reported to stabilize p53 and trigger cellular senescence [52]. Additionally, mTOR signaling is a major regulator of autophagy. Autophagy and senescence are closely related, but the relationship is not yet been fully understood. In mice with Braf V600E mutation and PTEN deficiency, the deletion of the autophagy-related gene Atg7 promotes melanoma formation by preventing senescence [53]. In cultured human fibroblasts, inhibition of the RAS effector pathway and activation of the autophagy-promoting PI3K-AKT signaling pathway lead to senescence through their effects on FOXO transcription factors [54]. mTORC1 actively inhibits autophagy by phosphorylating ULK1. Therefore, inhibition of mTORC1 induces autophagy to slow aging through the removal of damaged and dysfunctional mitochondria (mitophagy) [55]. Accumulating evidence indicates that the mTOR signaling pathway is critically involved in hematopoietic stem cell (HSC) senescence. Notably, pharmaceutical inhibition of mTOR improved the regenerative capacity of HSCs from aged mice, indicating that the functional capacity of these HSCs can be restored [56].

### Metabolic changes

**Fig. 1:**
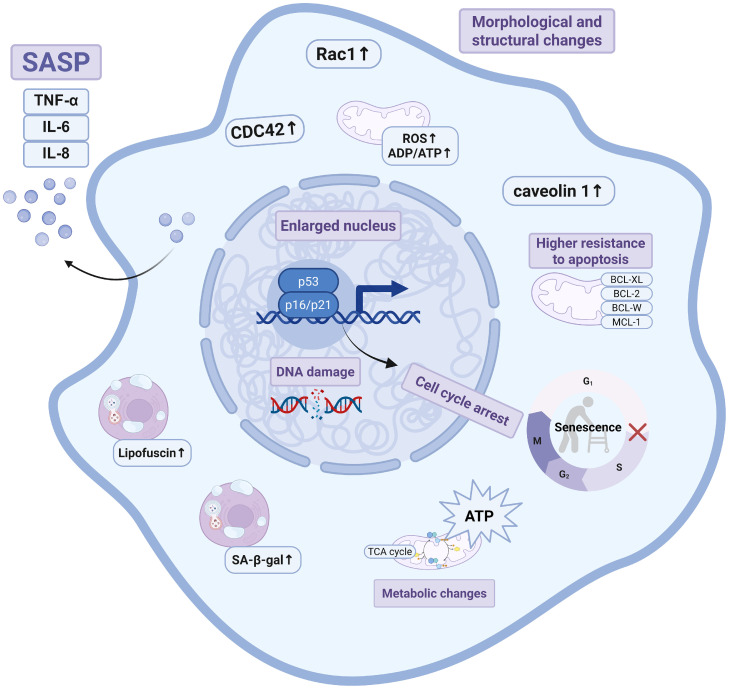
**Hallmarks of Cellular Senescence.** The characteristics of cellular senescence are closely related to morphological changes, increased SA-β-gal activity, increased lysosomal secretion of SASP-related factors, cell cycle arrest, oxidative stress regulation and DNA damage. SASP, Senescence‑associated secretory phenotype; IL-6: Interleukin‑6; IL-8: Interleukin‑8; TNF-α: Tumor necrosis factor‑α; CDC42: Cell division cycle 42; Rac 1: Ras-related C3 botulinum toxin substrate 1; SA-β-gal: Senescence‑associated β‑galactosidase; TCA: tricarboxylic acid; ROS: Reactive oxygen species; ATP: Adenosine triphosphate; ADP: Adenosine diphosphate; BCL-XL: B-cell lymphoma extra-large; BCL-2: B-cell lymphoma 2; BCL-W: B-cell lymphoma-W; MCL-1: Myeloid cell leukemia 1.

Senescent cells exhibit significant metabolic changes such as increased glycolysis, mitochondrial metabolism, and autophagy. Mitochondrial dysfunction may be a direct trigger of aging [57,58]. Senescent cells accumulate many mitochondria and typically exhibit respiratory chain defects and excessive production of reactive oxygen species (ROS). Various factors contribute to the induction of senescence, such as oxidative and proteotoxic stress, leading to protein misfolding and subsequent endoplasmic reticulum stress through the unfolded protein response [7,59]. Thus, senescent cells exhibit altered response to unfolded proteins [7]. Changes in lysosomes, mitochondria, and the endoplasmic reticulum may be both causes and consequences of senescence-associated metabolic alterations. In senescent cells, the ratios of AMP to ATP and ADP to ATP increase, indicating that senescent cells are high-metabolism cells [60]. Senescent cells utilize glycolysis, fatty acid oxidation, and oxidative phosphorylation in an abnormal manner to increase oxygen consumption [7,19,61]. Studies have reported that Rb activates pyruvate dehydrogenase, promoting the conversion of pyruvate to acetyl-CoA, which then enters the tricarboxylic acid (TCA) cycle, thereby increasing ATP production [61]. In addition, the SASP depends on an increase in ATP [7]. ATP promotes the SASP, leading to proteotoxic stress, which is alleviated by autophagy activation to mitigate the stress response (Fig. 1). The phenotypes, markers and detection methods are summarized in Table 1.

**Table 1:** Senescence phenotypes, markers and detection methods.

Phenotypes	Markers	Detection Methods
Morphological and structural changes	Nuclear and cellular enlargement Cellular flattening Lamin B1 Rac1 CDC42	LM SEM TEM WB
Cell cycle arrest	G1 phase arrest	FCM
	p53 p16 p21	WB IF
	Ki67 BrdU EdU	IHC IF
DNA damage	ATM ATR p-γH2AX 53BP1	WB IHC IF
SASP	IL-6 IL-8 TNF-α MMPs CXCLs	WB ELISA q-PCR
Lysosomal dependent changes	SA-β-gal Lipofuscin α-fucosidase	LM IF ELISA
Metabolic changes	ROS ATP	FCM IF ELISA

SASP, Senescence‑associated secretory phenotype; Rac 1: Ras-related C3 botulinum toxin substrate 1; CDC42: Cell division cycle 42; ATM, Ataxia-telangiectasia mutated; ATR, ATM and Rad3-related; 53BP1: p53-binding protein 1; IL-6: Interleukin‑6; IL-8: Interleukin‑8; TNF-α: Tumor necrosis factor‑α; MMPs: Matrix metalloproteinases; CXCLs: C-X-C motif chemokine ligands; SA-β-gal: Senescence‑associated β‑galactosidase; ROS, Reactive oxygen species; ATP: Adenosine triphosphate; LM: Light Microscope; SEM: Scanning electron microscope; TEM: Transmission electron microscope; WB: Western blot; FCM: Flow cytometry; IF: Immunofluorescence; IHC: Immunohistochemistry; ELISA: Enzyme-linked immunosorbent assay; q-PCR: Quantitative real-time polymerase chain reaction.

## Regulatory mechanisms of cellular senescence in hematologic malignancies

Increasing evidence has shown that cellular senescence plays an important role in the occurrence and development of various hematological malignancies. Cellular senescence in hematologic malignancies is orchestrated by a network of evolutionarily conserved signaling pathways, including p53/p21, p16/Rb, mTOR, TGF-β, and MAPK/NF-κB signaling. Here, we discuss the mechanisms and therapeutic implications of cellular senescence in hematological malignancies.

### Myelodysplastic syndromes (MDS)

MDS is a group of heterogeneous clonal stem cell disorders characterized by hematopoietic dysfunction, accompanied by varying degrees of cytopenia and dysplasia [62]. A study on aplastic anemia has indicated that telomere erosion accelerates hematopoietic stem cell senescence, potentially leading to secondary MDS [63]. Research has indicated that bone marrow mesenchymal stem cells (BM-MSCs) from MDS patients exhibit high expression of SASP factors, including IL-6 and tumor necrosis factor-alpha (TNF-α) [64]. In HSCs, increased ROS production and NLR family pyrin domain containing 3 (NLRP3) inflammasome activation may facilitate the onset of MDS [65]. MDS-derived S100A9 induces cellular senescence in MSCs by activating TLR4, the NLRP3 inflammasome and interleukin-1β (IL-1β) secretion [66]. Senescent BM-MSCs from MDS patients increase the survival of leukemia cells while decreasing the capacity for normal hematopoiesis [64,67]. Additionally, several studies have indicated that MSCs from MDS patients exhibit distinct senescence characteristics, such as altered cell morphology, reduced proliferation, changes in the cytoskeleton, and increased β-galactosidase activity, which may accelerate the transformation of MDS to acute myeloid leukemia (AML) [[68], [69], [70]]. Therefore, senescent MSCs may be potential targets for therapeutic intervention for MDS. Pretreating BM-MSCs from MDS patients with lenalidomide rescues impaired myeloid and erythroid colony formation in early hematopoietic progenitor cells [71]. TGF-β1 is considered a related driver of molecular alterations and senescence in MSCs. The TGFβ inhibitor SD-208 eliminates the suppressive effects of TGFβ1 on MSCs [72].

### Myeloid leukemia (ML)

ML is a hematologic malignancy that originates in the BM and affects mainly myeloid cells [73,74]. MDS can progress to AML, which is diagnosed when leukemic cells constitute ≥20 % of blood and BM infiltration, accompanied by cytopenia and genetic abnormalities such as t(8;21), inv(16), or t(16;16) [75]. BM-MSCs from AML patients show increased of SASP factors, including IL-6 [76], interleukin-7 (IL-7) [77], and CXCL8 [78]. The increased expression of SASP factors in the BM and plasma of MDS patients can help predict overall survival and progression to AML, supporting the potential role of senescence and SASP factors in the transition from MDS to AML. Notably, the gene expression of IL-6 in BM-MSCs gradually increases as MDS progresses to AML [75]. In AML, senescent MSCs participate in feedback through the SASP, promoting the survival and proliferation of AML cells. Therefore, the selective elimination of p16^INK4a+^ senescent MSCs in vivo increases the survival rate of leukemic mice [79], indicating the significant role of senescent BM-MSCs in AML. Additionally, the pathogenesis of AML is associated with abnormal differentiation and proliferation of clonal populations of BM stem cells, with hematopoietic stem cells undergoing senescence-associated epigenetic reprogramming [80]. mTOR activation is a fundamental cause of HSC senescence, and rapamycin treatment partially restores hematopoietic activity [81]. The transcription factor Spi1 is a key regulatory factor in hematopoiesis, that limits the self-renewal of hematopoietic stem cells. In hematopoietic cells, Spi1 overexpression induces cellular senescence, which may serve as a mechanism to prevent the development of AML [82]. In summary, senescent BM-MSCs accumulate with increasing age in MDS patients and may accelerate the progression to AML. Targeting MSCs may be a potential therapeutic approach for AML [83].

Mutant FLT3, particularly FLT3 internal tandem duplications (FLT3-ITD), is a driver oncogene in approximately 30 % of AML cases and is intricately linked to cellular senescence pathways [84]. Constitutively active FLT3-ITD signaling hyperactivates downstream effectors such as STAT5, MAPK, and PI3K/AKT/mTOR, which modulate senescence. A recent study has suggested that FLT3-ITD mutation suppresses p16^INK4a^ expression via the STAT5A-E2F3-EZH2 signaling axis. This downregulation of p16^INK4a^ allows cells to evade senescence, thereby promoting increased malignancy and establishing a positive feedback loop that exacerbates AML progression [85]. Understanding the crosstalk between FLT3 signaling and senescence is therefore critical for developing strategies to prevent relapse and eliminate persistent senescent-like leukemic cells.

Chronic ML (CML) leads to accelerated telomere shortening because of increased turnover of hematopoietic cells driven by the BCR-ABL oncogene [86]. Cell cycle arrest associated with senescent cells leads to slowed growth or halts proliferation. Studies have shown that the DNA demethylating agent 5-aza-2′-deoxycytidine (DAC) induces senescence in chronic leukemia cell lines, as evidenced by increased SA-β-gal activity and lysosomal mass, as well as by the upregulation of cell cycle-related genes. DAC reduces telomere length, telomerase activity, and human telomerase reverse transcriptase (hTERT) expression by inhibiting c-Myc binding to the hTERT promoter [87]. Previous studies have demonstrated that the vitamin D/VDR axis regulates DNA repair during oncogene-induced senescence [88]. BCR-ABL drives CML by requiring VDR, as indicated by VDR knockout inducing CML cell senescence through the DDIT4-mediated DDR signaling pathway [89].

### Lymphoid leukemia

Acute lymphoblastic leukemia (ALL) is the most common tumor occurring in children under 15 years of age. in vitro models of ALL, leukemia cells induce MSC senescence through ROS production and p53-mediated pathways, which promote senescence and support the proliferation of tumor cells [90]. Numerous studies have indicated that cellular senescence and β-Arrestin1 play critical roles in the survival of children with B-ALL [91,92]. The absence of β-Arrestin1 reduces the interaction between P300 and Sp1, which inhibits Sp1 binding to the hTERT promoter, downregulates hTERT transcription, decreases telomerase activity, and shortens telomere length, ultimately promoting ALL cell senescence [92]. The length of telomeres depends on telomerase activity, and potential drugs could be used to treat tumors by targeting telomeres and telomerase.

Chronic lymphocytic leukemia (CLL) is characterized by the clonal proliferation and accumulation of mature B cells (typically CD5 positive) in the blood, BM lymph nodes, and spleen [93]. Studies have shown that BM-MSCs isolated from CLL patients exhibit senescence characteristics, such as reduced CFU-F frequency [94], decreased growth rate [94], cell cycle arrest, increased polygonal morphology [95], elevated β-galactosidase staining [95], and increased p16^INK4a^ mRNA expression [95]. Additionally, MSCs in the BM and peripheral blood of CLL patients show increased expression of SASP factors, including IL-6, CXCL8, and vascular endothelial growth factor (VEGF) [[95], [96], [97]]. Moreover, CLL patients with elevated serum levels of IL-6, TNF-α, and CXCL8 have lower overall survival rates [[98], [99], [100]]. Studies have indicated that the levels of IL-6, CXCL8, and TNF-α in the peripheral blood of CLL patients are positively correlated with patient age [98,100]. However, whether the elevated levels of these cytokines are associated with the age-related accumulation of senescent MSCs remains unclear. Notably, exogenous CXCL8 prolongs leukemia cell survival in vitro by upregulating the expression of antiapoptotic proteins in B-CLL cells [101]. Because IL-6, VEGF, and CXCL8 are involved in the SASP, the age-related accumulation of senescent BM-MSCs may provide a microenvironment for CLL growth through paracrine proliferation and growth factors. Currently, whether senescent BM-MSCs promote the growth of clonal B cells in CLL patients is unclear, and further studies are needed to clarify the role of senescent MSCs in CLL.

### Lymphoma

Lymphoma includes Hodgkin lymphoma (HL) and non-Hodgkin lymphoma (NHL), Reed-Sternberg (RS) cells in Hodgkin lymphoma have a distinctive large cell morphology, which is crucial for the diagnosis of HL [102]. RS cells have characteristics of senescent cells, including high expression of p16^INK4a^ and p21^Cip1^, and oxidative stress promotes senescence in RS cells [103]. Additionally, a previous study has revealed that p62 is highly expressed in RS cells and may serve as a prognostic biomarker for classical HL (cHL) [104]. The NF-κB signaling pathway is activated in RS cells, and NF-κB is a regulator of the SASP, which promotes the production of inflammatory cytokines [103]. The senescence of RS cells may facilitate interactions with other immune cells and remodel the HL microenvironment, thereby supporting tumor survival and proliferation. Inhibition of NF-κB and IL-6 has been used to treat HL. Curcumin alleviates symptoms of senescence [105], and both curcumin and the NF-κB inhibitor JSH reduce the secretion of IL-6 [103]. Notably, the use of proteasome inhibitor bortezomib as an NF-κB inhibitor for the treatment of HL in the clinic has failed [106,107]. However, other alternative NF-κB inhibitors have the potential to successfully treat HL. Additionally, studies have shown that the senescence of RS cells is associated with telomere shortening [108]. Among the genes coding for the MYST family of lysine acetyltransferases (KATs) are the KAT6A (also known as MOZ) and KAT6B (also known as MORF and QKF) oncogenes. Loss of one allele of KAT6A extends the median survival of mice with MYC-induced lymphoma. Selective inhibitors of KAT6A KAT6B, WM-8014 and WM-1119 induce cell cycle exit and cellular senescence without causing DNA damage. Senescence is INK4A/ARF-dependent and is accompanied by changes in gene expression that are typical of loss of KAT6A function [109]. In summary, inducing cell cycle exit and senescence is effective in preventing the progression of lymphoma. Targeting key molecules involved in senescence to develop drugs is essential for improving improve the treatment of lymphoma.

### Multiple myeloma

Multiple myeloma (MM) is characterized by M protein levels ≥30 g/L and/or monoclonal plasma cells ≥10 % in the BM, and it is typically accompanied by hypercalcemia, renal failure, anemia, and bone lesions [110]. In MM patients, BM-MSCs exhibit a senescent phenotype, including increased cell volume, reduced proliferative potential, decreased osteogenic differentiation potential, elevated levels of p16^INK4a^ and p21^Cip1^, increased β-galactosidase activity, and decreased expression of Dicer1 and cell cycle-related genes [70,[111], [112], [113], [114]]. Additionally, the expression of SASP factors, including IL-6, TNF-α, growth differentiation factor 15 (GDF15), and CXCL8, is significantly increased in the BM-MSCs of MM patients [92,111,112,114,115].Notably, in newly diagnosed MM patients, elevated serum levels of IL-6 and GDF15 are significantly associated with shortened overall survival [116,117]. Therefore, the levels of SASP factors are negatively correlated with the survival rate of MM patients and are associated with disease severity and progression. It remains unclear whether the increase in serum SASP factors is related to the accumulation of senescent BM-MSCs. However, there is a connection between BM-MSC senescence and the outcomes of MM. Additionally, myeloma cells stimulate MSCs to produce the enzymes required for LPA biosynthesis, with LPA1 and LPA3 transmitting opposing signals to MSCs, thereby determining their fate. LPA3 activation increases the expression of antioxidant enzymes, inhibits the accumulation of ROS, and alleviates cellular senescence. Silencing LPA3 in MSCs in vitro is associated with cellular senescence and significantly promotes the progression of MM and tumor-associated angiogenesis in vivo. In contrast, siLPA1-MSCs resist cellular senescence in vitro and effectively delay the progression of MM [118,119]. Therefore, LPA signaling may be a potential therapeutic target for MM. Additionally, CD8^+^CD28^-^ T cells expressing CD57 accumulate in the BM of MM patients, suggesting that these cells may be ineffective for tumor immune surveillance [120]. Oncogene-induced senescence (OIS) is a senescence program driven by activated oncogenes and acts as a cellular tumor-suppressive mechanism in various precancerous lesions. Compared with MM, smoldering MM (SMM) is characterized by the overexpression of biomarkers of cellular senescence, supporting the hypothesis that OIS serves as a breakpoint mechanism against malignant transformation in plasma cell disorders [121]. Doxorubicin treatment enriches miR-433 in exosomes, which in turn induces malignant senescence in MM cells. Studies have revealed that the establishment of a senescent phenotype in neighboring MM cells is p53- and p21-independent and is related to CDK-6 downregulation [122].

## Therapeutic strategies targeting cellular senescence

### Inducing cellular senescence

Cellular senescence is an important barrier to tumor initiation and progression, and “senescence-inducing” therapies are promising strategies for cancer treatment. In recent years, several CDK4/6 inhibitors, such as abemaciclib and palbociclib, have been approved by the Food and Drug Administration (FDA). These senescence-inducing compounds can be used in clinical chemotherapy for various malignancies, including breast cancer and non-small cell lung cancer, and they have promising therapeutic effects [123]. Combination therapy involving CDK4/6 inhibitors may target multidrug resistance in hematologic malignancies [124]. There is growing evidence that the p53/MDM2 axis is critically involved in the regulation of aging, senescence and oncogenesis [125]. A phase Ib trial has demonstrated that venetoclax-idasanutlin treatment exhibits manageable safety and encouraging efficacy in unfit patients with relapsed/refractory (R/R) AML [126]. Clinical activity of AMG232, an investigational oral, selective MDM2 inhibitor, has been observed in some patients. However, the maximum tolerated dose (MTD) of AMG232 has not been reached because of gastrointestinal AEs at higher doses [127]. AMG232 plus dabrafenib (D) ± trametinib (T) exhibits a favorable PK profile in MM [128]. RG7112, another small-molecule MDM2 antagonist, has demonstrated clinical activity against R/R AML and CLL/sCLL [129] (Table 2). Senescence-inducing strategies have also shown promising applications in the treatment of hematologic malignancies. Several studies have demonstrated that the induction of cellular senescence mediates CML treatment. For instance, 4-methylumbelliferone (4MU) and the small molecule Aurora inhibitor AKI603 improve drug resistance in CML. 4MU induces cellular senescence by inhibiting hyaluronic acid synthesis, ultimately reversing resistance to vincristine in CML cell lines [130]. AKI603 overcomes drug resistance induced by the BCR-ABL-T315I mutation in CML and induces senescence in both wild-type BCR-ABL and T315I-mutant leukemia cells. This induction of leukemia cell senescence may be associated with increased ROS levels [131]. Research has shown that ACSL1 promotes imatinib-induced senescence in CML cells by regulating the SIRT1/p53/p21 pathway [132]. Additionally, MRK-107 reduces cell proliferation and increases cellular senescence by increasing lipid peroxidation and lowering glutathione (GSH) levels, thereby inducing oxidative stress in tumor cells. Thus, the induction of cellular senescence by MRK-107, along with oxidative stress, may be a potential mechanism underlying its antitumor effects against CML [133]. The antitumor mechanisms that induce cellular senescence are also significant in acute leukemia. Previous studies have shown that MYC effectively induces apoptosis. CDK2 inhibitors suppress MYC/BCL-XL-driven AML in mice by inducing cellular senescence [134]. Additionally, the BRAF inhibitor vemurafenib (VEM) significantly induces senescence, inhibits proliferation, and promotes apoptosis in AML cells. Furthermore, bortezomib (Bortezomib) enhances these effects, potentially through the activation of the HIPPO signaling pathway by the VEM-BOR combination, which induces senescence in AML cells and xenograft mice [135]. Erythropoietin (EPO) suppresses p53-dependent apoptosis induced by genotoxic (daunorubicin, doxorubicin, and γ-radiation) and nongenotoxic (nutlin-3a) agents, and it induces a senescence-like state in myeloid leukemia cells. EPO interferes with stress-dependent Mdm2 downregulation and leads to destabilization of the p53 protein. Furthermore, EPO selectively modulates the expression of p53 target genes in response to DNA damage, preventing the induction of several noncoding RNAs (ncRNAs) previously associated with p53-dependent apoptosis [136]. Additionally, studies have shown that the Aurora A kinase inhibitor MLN8237 induces cellular senescence and inhibits the proliferation of MM cells by upregulating p53 and the tumor suppressor genes p21 and p27 [137].

**Table 2:** Current clinical trials involving senolytics in hematologic malignancies.

Targeted molocular/pathway	Drug name	Disease	Clinical trials	Phase	Outcomes	References
BCL-2/BCL-XL/BCL-W/MCL-1	ABT-263 (Navitoclax)	R/R ALL and LL	NCT03181126	Phase I	Venetoclax with low-dose navitoclax and chemotherapy was well tolerated and had promising efficacy	[144]
		CLL	NCT00481091	Phase I	Navitoclax dose of 250 mg/d in a continuous dosing schedule was optimal for phase II studies	[146]
		LM	NCT00406809	Phase I	150 mg 7-day lead-in dose followed by a 325 mg dose administered on a continuous 21/21 dosing schedule was selected for phase II study	[145]
		CLL	NCT01087151	Phase II	N/A	N/A
	S63845/S64315 (MIK665)	AML/MDS	NCT02979366	N/A	N/A	N/A
MDM2	Idasanutlin (RG7388)	R/R AML	NCT02670044	Phase I	IDH1/2 and RUNX1 mutations were associated with venetoclax-idasanutlin sensitivity	[126]
	AMG232	AML	NCT02016729	Phase I	The MTD of AMG 232 was not reached. Dose escalation was discontinued due to GI AEs at higher doses	[127]
		MM	NCT03031730	Phase I	The maximum tolerated dose of AMG 232 was 120 mg; adding AMG 232 to trametinib±dabrafenib did not confer additional clinical benefit	[128]
	RG7112	R/R AML and CLL/sCLL	NCT00623870	Phase I	High dose RG7112 required for efficacy leading to GI intolerance and variability of exposure at the MTD	[129]
TKI	Dasatinib	R/R Leukemia	NCT00306202	Phase I	Dasatinib 60 mg/m^2^ and 80 mg/m^2^ once-daily dosing were selected for phase II studies in children with pH^+^ leukemia	[150]
		pH^+^ALL	NCT01460160	Phase II	-	-
		pH^+^ALL	NCT00720109	Phase III	-	-

**Fig. 2:**
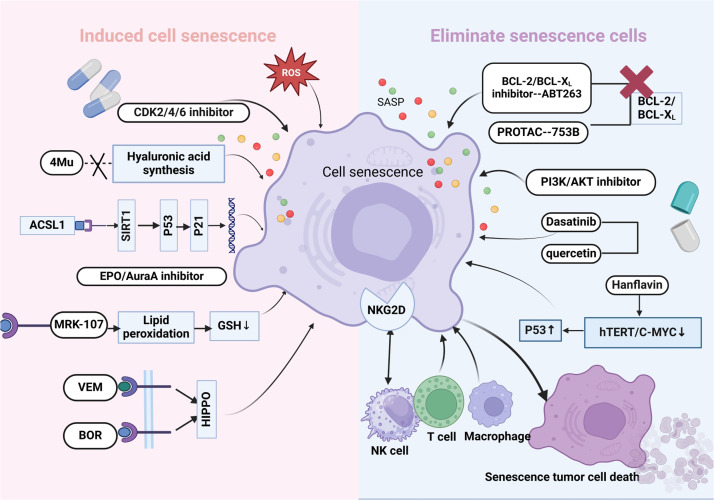
**Cellular senescence in hematologic malignancies: potential mechanism for precision therapeutics.** CDK: Cyclin dependent kinase; 4Mu: 4-Methylumbelliferone; ACSL1: Acyl-CoA synthetase long chain family member 1; SIRT1: Sirtuin 1; EPO: Erythropoietin; AuraA: Aurora kinase A; GSH: Glutathione; VEM: Vemurafenib; BOR: Bortezomib; ROS: Reactive oxygen species; SASP: Senescence‑associated secretory phenotype; BCL-2: B-cell lymphoma 2; BCL-XL: B-cell lymphoma extra-large; PI3K/AKT: Phosphatidylinositol 3-kinase / Protein Kinase B; hTERT: Human telomerase reverse transcriptase; C-MYC: Cellular myelocytomatosis viral oncogene homolog; NKG2D: Natural Killer Group 2, Member D.

The induction of cellular senescence as an antitumor strategy has significant clinical implications in hematological malignancies, and it may provide new ideas and methods for the treatment of hematological malignancies through mechanisms such as inhibition of tumor cell proliferation, induction of oxidative stress, and improved drug resistance (Fig. 2).

### Eliminating senescent cells

Although inducing senescence in cancer cells represents a promising therapeutic strategy, its clinical application requires careful consideration of its “double-edged sword” nature. The timing and duration of senescence induction are critical determinants of its safety and efficacy. In early-stage or premalignant conditions (e.g., low-risk MDS and SMM), OIS may serve as a natural barrier against malignant transformation [138]. Therefore, therapeutic induction of senescence could stabilize the disease or delay disease progression. However, in advanced or R/R diseases, prolonged senescence—especially when accompanied by a proinflammatory SASP—can promote a tumor-promoting microenvironment, facilitating immune evasion, therapy resistance, and disease recurrence [139]. Moreover, senescence in normal hematopoietic or stromal cells may contribute to secondary malignancies. Aging human HSCs exhibit profound epigenetic reprogramming of enhancers that may predispose them to leukemia [140]. AML relapse is facilitated by a senescence-like resilient phenotype that occurs regardless of stem cell status [141]. Therefore, patient stratification based on disease stage and SASP profiling is essential.

Currently, relevant preclinical studies focusing on the elimination of senescent cells including tumor cells and other cells, in the hematopoietic microenvironment have been reported. BCL-2 and BCL-XL have antiapoptotic effects and are highly expressed in both tumor cells and senescent cells [142]. The use of the BCL-2 and BCL-XL specific inhibitor ABT-263 effectively eliminates naturally aged or irradiation-induced prematurely senescent muscle stem cells and HSCs in C57BL/6 mice by inducing apoptosis, thereby restoring the vitality of these aged cells [143]. A phase I study has reported that venetoclax with low-dose ABT-236 is well tolerated and has promising efficacy in patients with R/R ALL or LL [144]. Preliminary clinical activity of navitoclax was observed in parallel phase I trials involving patients with lymphoma [145] and CLL [146]. A phase II clinical study on the effect of ABT-263 on CLL is ongoing. S64315, an MCL-1 inhibitor, has been explored in patients with AML or MDS (NCT02979366) (Table 2). 753B, as a novel PROTAC, exhibits senolytic activity by enhancing chemotherapy efficacy through the induction of cellular senescence and degradation of the antiapoptotic proteins BCL-XL and BCL-2, demonstrating strong potential in FLT3-ITD AML [147].

Additionally, PI3K/AKT inhibitors induce apoptosis in tumor cells, while dasatinib primarily targets and eliminates senescent adipocytes. Moreover, quercetin clears senescent BM-MSCs and endothelial cells. Research has suggested that compared with their individual use, a combination of these drugs more effectively targets a broader range of senescent cell types [148]. Studies have shown that compared with control treatment, treatment of C57BL/6 mice with dasatinib and quercetin significantly reduces the expression levels of p16^INK4a^ mRNA in osteocytes and decreases the percentage of senescent osteocytes [149]. Current clinical data support the rationale for continued dasatinib development in pediatric patients with pH^+^ leukemia [150]. Pediatric trials currently underway include phase II (NCT01460160) and III (NCT00720109) trials investigating the use of dasatinib in combination with multiagent chemotherapy for pH^+^ ALL. Most current clinical studies on senolytics are still in phase I/II and more clinical studies need to explore the application of senolytics in hematological malignancies (Table 2). Moreover, hesperetin, a natural flavonoid, has significant antitumor effects on T-cell malignancies. Hesperetin induces cellular senescence by inhibiting the transcriptional activity of hTERT and c-MYC and activating the p53-mediated DNA damage response. The combination of the BCL-2 inhibitor navitoclax with hesperetin significantly increases the apoptosis rate of senescent cells, enhancing the therapeutic effects on tumors with low BCL-2 expression [151].

The targeted clearance of senescent cells is driven by various immune cells, such as cytotoxic T cells, macrophages, and natural killer (NK) cells, through a process known as senescence surveillance [152,153]. NK cells exhibit high cytotoxicity against tumor cells, and their ability to kill senescent cells is regulated by ligands on senescent cells. NKG2D ligands located on the cell membrane interact with NK cells and mediate the effective killing of senescent cells [154,155]. Allogeneic or genetically modified NK cell infusion is currently considered an effective therapeutic approach for hematologic malignancies. In preclinical studies, infusion of expanded NK cells has been shown to be a feasible and safe method for patients with various types of hematologic malignancies [156]. In summary, these studies demonstrate the significant potential of strategies targeting the clearance of senescent cells in hematologic malignancies, providing new directions and insights for the treatment of these conditions (Fig. 2).

BCL-XL: B-cell lymphoma extra-large; BCL-2: B-cell lymphoma 2; BCL-W: B-cell lymphoma-W; MCL-1: Myeloid cell leukemia 1; MDM2: Murine double minute 2 homolog; TKI: Tyrosine kinase inhibitor; R/R: Relapsed/Refractory; ALL: Acute lymphoblastic leukemia; LL: lymphoblastic leukemia; CLL: Chronic lymphoblastic leukemia; LM: Lymphoid malignancies; AML: Acute myeloid leukemia; MDS: Myelodysplastic syndromes; MM: Multiple myeloma; sCLL: Small cell lymphocytic lymphoma; IDH1/2: Isocitrate dehydrogenase 1/2; RUNX1: Runt-related transcription factor 1; MTD: Maximum tolerated dose; GI: gastrointestinal; AEs: Adverse events; pH^+^: Philadelphia chromosome positive

## Summary and prospect

Research related to cellular senescence has shown tremendous potential in the treatment of hematologic malignancies. By inducing cellular senescence and eliminating senescent cells, these strategies inhibit tumor growth and increase the efficacy of chemotherapy. Combining current antitumor treatments and senolytics can eliminate senescent cells more effectively, thereby improving treatment outcomes. A deeper understanding of the biological characteristics of senescent cells and their roles in the tumor microenvironment will provide crucial insights for the development of new therapeutic strategies. Furthermore, exploring personalized treatment approaches that incorporate the role of immune cells may open new avenues for therapy, further improving the prognosis of hematologic malignancies.

## CRediT authorship contribution statement

**Peijie Jiang:** Writing – original draft. **Guancui Yang:** Investigation. **Jiarun Li:** Investigation. **Xiaolong Tian:** Investigation. **Xueqing Yang:** Investigation, Conceptualization. **Shijie Yang:** Conceptualization. **Jin Wei:** Data curation. **Xi Zhang:** Writing – review & editing. **Jinyi Liu:** Writing – review & editing, Funding acquisition.

## Declaration of competing interest

The authors declare that there is no conflict of interests. All authors have reviewed the content in full and agreed on submission.

## Appendix Supplementary materials

Image, application 1
